# Efficacy and safety outcomes in Japanese patients with low-risk polycythemia vera treated with ropeginterferon alfa-2b

**DOI:** 10.1007/s12185-024-03804-1

**Published:** 2024-07-01

**Authors:** Kazuya Shimoda, Albert Qin, Norio Komatsu, Keita Kirito

**Affiliations:** 1 Division of Hematology, Diabetes and Endocrinology, Department of Internal Medicine, Faculty of Medicine, University of Miyazaki, 5200 Kihara, Kiyotake‑cho, Miyazaki‑city, Miyazaki 889‑1692 Japan; 2 Medical Research & Clinical Operations, PharmaEssentia Corporation, 13F, No. 3, Park Street, Nangang District, Taipei, 115 Taiwan; 3 PharmaEssentia Japan KK, Akasaka Center Building 12F, 1‑3‑13 Moto‑akasaka, Minato‑ku, Tokyo 107‑0051 Japan; 4 Department of Hematology, Juntendo University Graduate School of Medicine, 2‑1‑1 Hongo, Bunkyo‑ku, Tokyo 113‑8421 Japan; 5 Department of Advanced Hematology, Juntendo University Graduate School of Medicine, 2‑1‑1 Hongo, Bunkyo‑ku, Tokyo 113‑8421 Japan; 6 Department of Hematology and Oncology, University of Yamanashi, 1110 Shimokato, Chuo‑shi, Yamanashi 409‑3898 Japan

**Keywords:** Hematologic response, *JAK2* V617F allele burden, Low-risk, Polycythemia vera, Ropeginterferon alfa-2b

## Abstract

Polycythemia vera (PV) is a Philadelphia chromosome-negative myeloproliferative neoplasm characterized by clonal erythrocytosis. A phase 2 study reported that ropeginterferon alfa-2b is a well-tolerated and effective treatment for PV in Japanese patients. This post hoc analysis of the phase 2 data further evaluated outcomes in patients at low risk of thrombosis (low-risk PV). Among 20 patients with low-risk PV, 60.0% (12/20) and 85.0% (17/20) achieved < 45% hematocrit by weeks 24 and 52, respectively. The proportion of responders with complete hematologic response (CHR) was 60.0% (12/20) at week 52, and the median time to response was 11.9 months. The mean *JAK2* V617F allele burden decreased from 75.8% at baseline to 53.7% at week 52. No patient experienced thrombosis or bleeding episodes. All patients experienced treatment-emergent adverse events (TEAEs) related to ropeginterferon alfa-2b, but no grade ≥ 3 TEAEs or deaths related to ropeginterferon alfa-2b occurred, and no new safety concerns arose. This analysis indicated that ropeginterferon alfa-2b may be an effective treatment option for Japanese patients with low-risk PV.

**Supplementary Information:**

The online version contains supplementary material available at 10.1007/s12185-024-03804-1.

## Introduction

Patients with polycythemia vera (PV) have an overproduction of erythrocytes, and lowering hematocrit (HCT) levels < 45% is recommended to reduce the risk of thrombotic events (TEs) and cardiovascular death [1]. The National Comprehensive Cancer Network and the European LeukemiaNet recommend cytoreductive therapy (CRT) for patients with PV (including those at low risk of thrombosis [low-risk PV], e.g., patients < 60 years old without previous TEs) who have either poor hematologic control, symptomatic PV, splenomegaly, frequent phlebotomies or are intolerant to phlebotomy [2, 3]. Currently, Japanese guidelines only recommend phlebotomy and the use of low-dose aspirin for low-risk PV [4]. However, evidence suggests that persistently elevated hematologic parameters, including HCT, white blood cell (WBC) and platelet (PLT) counts, and higher *JAK2* V617F allele burden can increase the risk of TEs even in patients with low-risk PV [5–7]. Thus, patients with low-risk PV require proper management of hematologic parameters and *JAK2* V617F allele burden.

Ropeginterferon alfa-2b is a novel, site-selective, monopegylated recombinant human interferon alfa-2b that is a well-tolerated and effective treatment for PV [8, 9]. The LOW-PV study reported that ropeginterferon alfa-2b is safe and effective in patients with low-risk PV [10]. However, no such evidence has been reported in Japanese patients. This analysis of a phase 2 study evaluated safety and efficacy of ropeginterferon alfa-2b in Japanese patients with low-risk PV.

## Materials and methods

This was a post hoc analysis of a phase 2, open-label, multicenter, single-arm study in Japanese PV patients [9]. Patients (aged ≥ 20 years and with a diagnosis of PV [11, 12]) received subcutaneous ropeginterferon alfa-2b every 2 weeks for 12 months, starting at 100 µg (50 µg for patients already receiving CRT) and up to a maximum dose of 500 µg [9].

This analysis evaluated data from patients with low-risk PV (age < 60 years and no history of thrombosis) [4]. The key inclusion criteria are shown in Supplemental Table 1.

HCT, WBCs, PLTs, and *JAK2* V617F allele burden were analyzed. The complete hematologic response (CHR) maintenance rate was defined as a HCT value < 45%, WBC count ≤ 10 × 10^9^/L, and PLT count ≤ 400 × 10^9^/L. Treatment-emergent adverse events (TEAEs) and their severity according to the Common Terminology Criteria for Adverse Events version 5.0 were assessed. SAS version 9.4 or higher (SAS Institute Inc., Cary, NC, USA) was used to generate figures and analyze data. The study was conducted in compliance with the ethical principles originating with the Declaration of Helsinki and all other relevant guidelines and requirements.

## Results

Twenty patients with low-risk PV were analyzed (median age: 49.5 years; Table 1). Median HCT, WBC count, and PLT count were 48.1%, 16.7 × 10^9^/L, and 798 × 10^9^/L, respectively. Poor baseline hematologic control was observed: 17/20 (85.0%) patients had a HCT ≥ 45%, 12/20 (60.0%) had a WBC count ≥ 15.0 × 10^9^/L, and 6/20 (30.0%) had a platelet count ≥ 1,000 × 10^9^/L. One patient was negative for the *JAK2* V617F variant; the mean *JAK2* V617F allele burden was 75.8% in the remaining 19 patients. Eight (40%) patients had received hydroxyurea treatment.

**Table 1:** Baseline demographics of patients with low-risk polycythemia vera

Baseline characteristic	*N* = 20
Age, years	49.5 (26–59)
Female sex	11 (55.0)
Hematocrit, %	48.1 (40.5–55.4)
White blood cell count, 10^9^/L	16.7 (6.1–33.4)
Platelet count, 10^9^/L	798 (356–1781)
Prior hydroxyurea treatment	8 (40.0)
Aspirin use	14 (70.0)
*JAK2* V617F mutation	19 (95.0)
*JAK2* V617F allele burden, %	75.8 ± 19.9

Data are median (range), *n* (%), or mean ± standard deviation

**Fig. 1:**
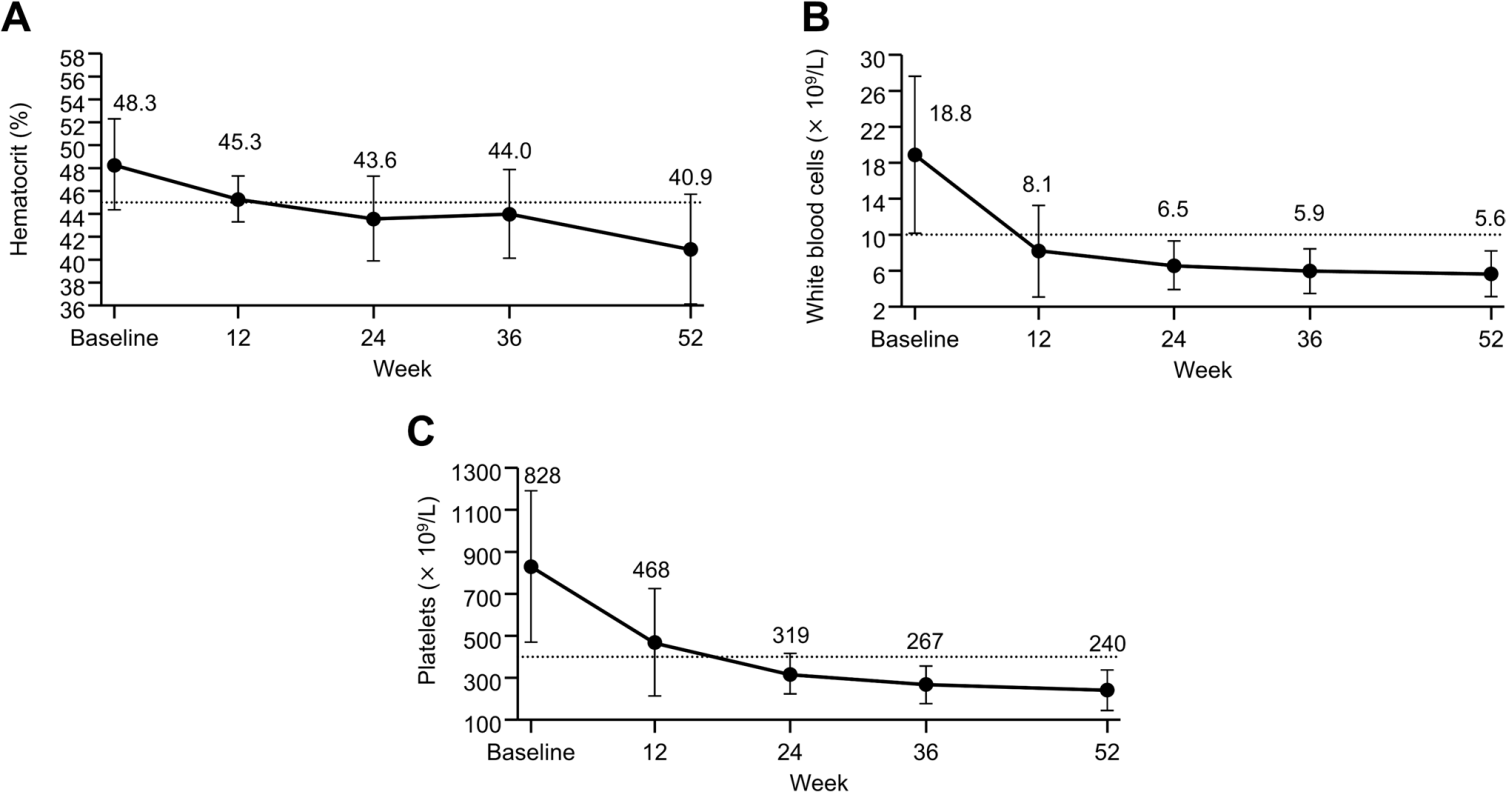
Laboratory values. **A** Hematocrit. **B** White blood cell count. **C** Platelet count. Data are mean (standard deviation). The dotted line indicates the target value (hematocrit: 45%, platelets: 400 × 10^9^/L, white blood cells: 10 × 10^9^/L)

HCT, WBC count, and PLT count decreased over the treatment period (Fig. 1). Most patients had < 45% HCT at weeks 24 (60.0% [12/20]) and 52 (85.0% [17/20]). The proportions of patients requiring phlebotomy were 35.0% (7/20) at baseline and 5.0% (1/20) at week 52 (Supplemental Fig. 1).

**Fig. 2:**
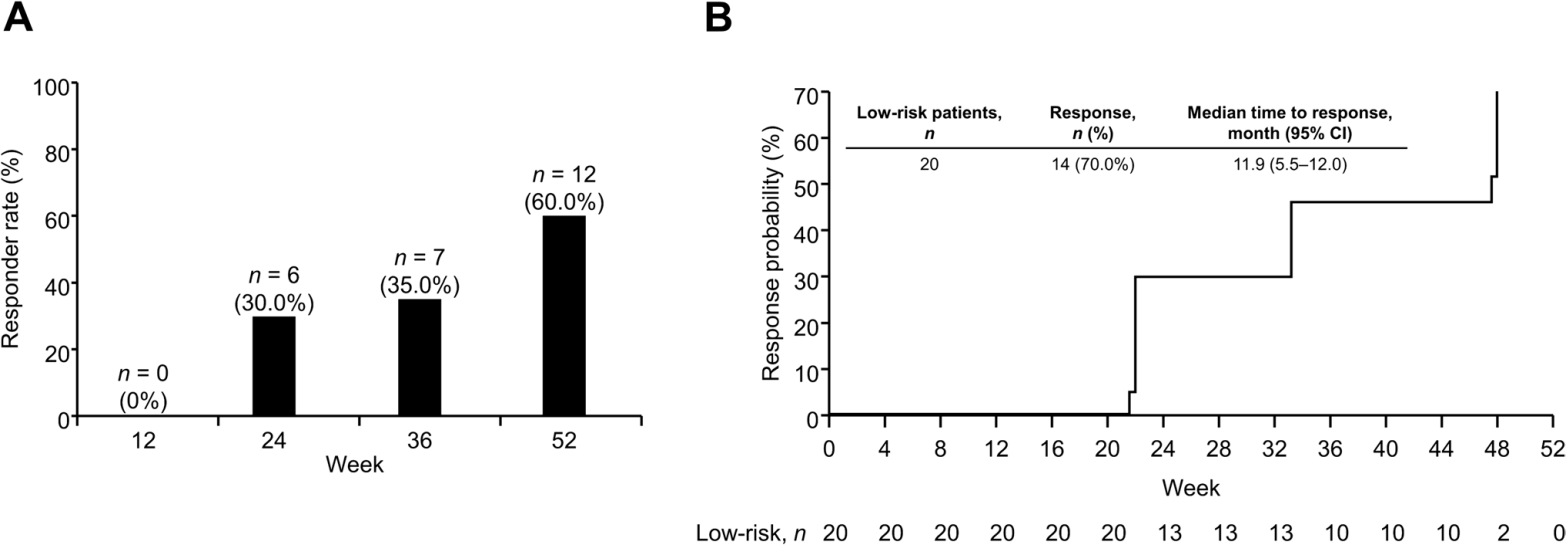
Complete hematologic response. **A** Proportion of responders with low-risk polycythemia vera over time. **B** Time to response. *CI* confidence interval

**Fig. 3:**
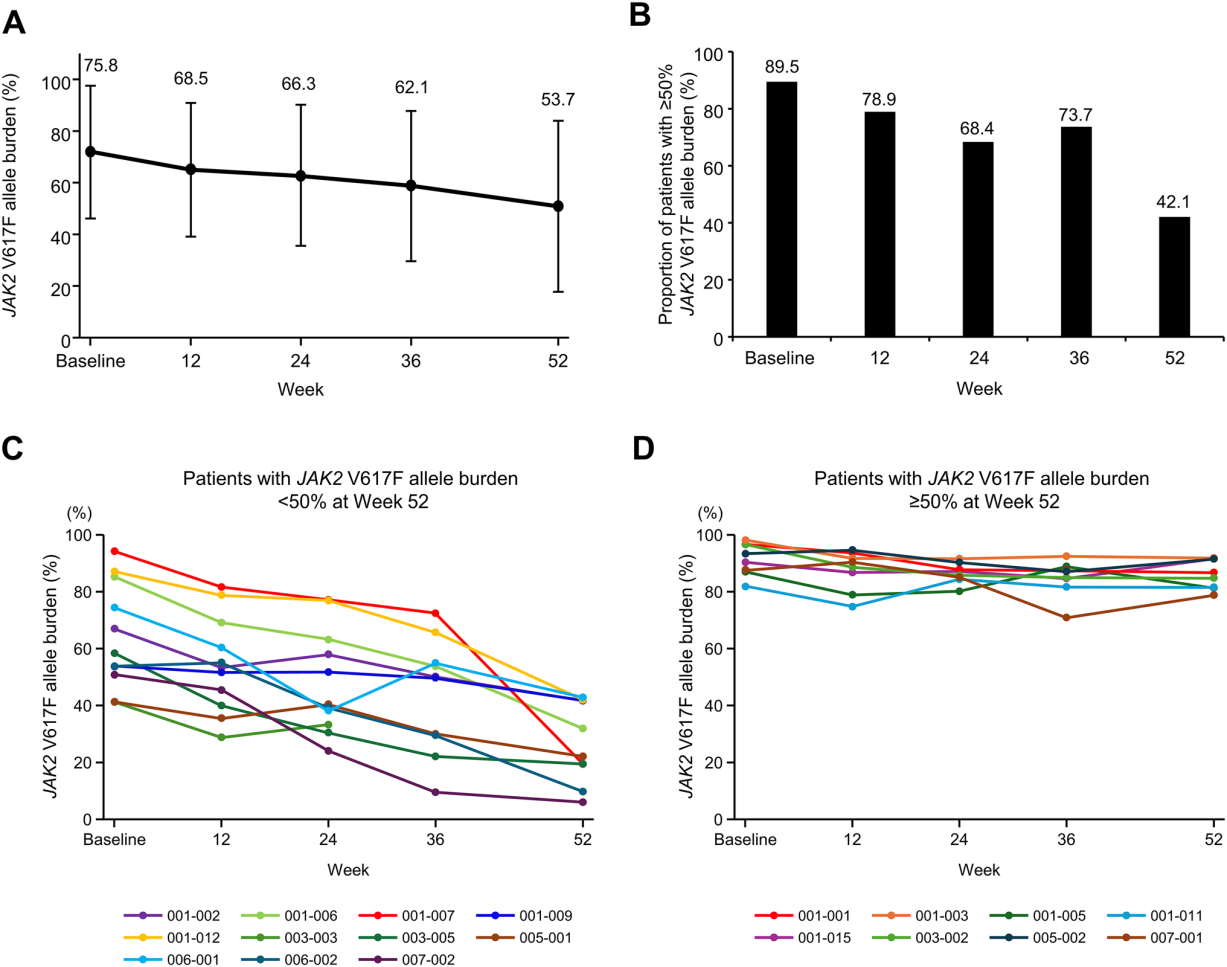
Molecular response. **A** *JAK2* V617F allele burden. **B** Proportion of patients with ≥ 50% *JAK2* V617F allele burden. **C** Change in *JAK2* V617F allele burden over time for patients with < 50% allele burden at week 52. One patient (003–003) discontinued, whose data at week 24 reflect the end of treatment. **D** Change in *JAK2* V617F allele burden over time for patients with ≥ 50% allele burden at week 52. *N* = 19; data are mean (standard deviation) or percentage. One patient who was negative for *JAK2* V617F at baseline was excluded

The proportions of patients with CHR were 30.0% (6/20) and 60.0% (12/20) at weeks 24 and 52, respectively; median time to response was 11.9 months (Fig. 2). The mean *JAK2* V617F allele burden decreased from 75.8% at baseline to 53.7% at week 52 (Fig. 3A). The proportion of patients with ≥ 50% variant allele frequency decreased from 89.5% (17/19) at baseline to 42.1% (8/19) at week 52 (Fig. 3B), although two patients had a < 50% of variant allele at baseline. Changes in *JAK2* V617F allele burden over time according to patients with < 50% or ≥ 50% allele burden at week 52 are shown in Fig. 3C and D, respectively. The patients who achieved < 50% allele burden at week 52 (11/19 cases, including 2 patients with allele burden < 50% at baseline) had a mean age of 50 years and mean allele burden of 64.4% at baseline; while those who did not achieve < 50% allele burden at week 52 (8/19 cases) had a mean age of 48 years and mean allele burden of 91.5% at baseline. Furthermore, the proportion of patients with CHR at week 52 was 100% (10/10 cases; one case could not be determined at week 52 due to discontinuation, but CHR was not achieved at end of treatment) in patients who achieved < 50% allele burden at week 52 and 37.5% (3/8 cases) in those who did not. The mean dose of ropeginterferon alfa-2b at week 52 was 350.0 μg in patients who achieved < 50% allele burden and 462.5 μg in those who did not.

TEAEs related to ropeginterferon alfa-2b occurred in all patients; however, no deaths or grade ≥ 3 TEAEs related to ropeginterferon alfa-2b occurred (Supplemental Table 2). The most common TEAEs observed in the low-risk group but not in the high-risk group were alanine aminotransferase increased (*n* = 6, 30.0%), aspartate aminotransferase increased (*n* = 5, 25.0%), and diarrhea (*n* = 5, 25.0%). One patient discontinued treatment because of grade 1 silent thyroiditis related to ropeginterferon alfa-2b [13]. One serious TEAE (gastroenteritis) occurred; this was not related to ropeginterferon alfa-2b.

## Discussion

This post hoc analysis of a phase 2 study found that ropeginterferon alfa-2b elicited a reduction in HCT values, WBC counts, PLT counts, and *JAK2* V617F allele burden in Japanese patients with low-risk PV.

As patients with low-risk PV for whom the standard treatment in Japan is difficult to apply (including patients for whom CRT is recommended because of disease-related signs and symptoms) were enrolled in the original phase 2 study [2, 3, 9], the patients in this analysis exceeded target hematological levels at baseline, and exhibited HCT: 48.3%, WBC: 18.8 × 10^9^/L, and PLT: 828 × 10^9^/L. Lowering HCT levels < 45% is recommended to prevent TEs [4], and elevated WBC and PLT counts are also risk factors for TEs [5, 6]. At 52 weeks, the mean values reached the target value (HCT: 40.9%, WBC: 5.6 × 10^9^/L, PLT: 240 × 10^9^/L), and no TEs occurred, indicating the usefulness of ropeginterferon alfa-2b in low-risk patients with PV.

In the LOW-PV study (which compared standard treatment [phlebotomy and low-dose aspirin] versus standard treatment plus ropeginterferon alfa-2b for low-risk PV), the percentage of responders (HCT < 45% and no disease progression) at 12 months was 81% in the ropeginterferon alfa-2b group [10]. Furthermore, there were fewer mean phlebotomies per patient-year in the ropeginterferon alfa-2b group (2.9) than the standard treatment group (4.2). The percentage of patients who achieved HCT < 45% in our study (85%) was similar to that of LOW-PV. Similarly, fewer patients required phlebotomies following ropeginterferon alfa-2b treatment, indicating consistent outcomes in the Japanese population.

In this analysis, the mean *JAK2* V617F allele burden notably decreased, and the proportion of patients with a ≥ 50% variant allele frequency decreased from 89.5% at baseline to 42.1% following 52 weeks of treatment. Furthermore, no cases experienced myelofibrosis transformation. A previous study found that patients with higher *JAK2* V617F burden (> 50% variant allele frequency) have a higher myelofibrosis transformation rate than patients with < 50% frequency [14]. Therefore, the decreased *JAK2* V617F allele burden following ropeginterferon alfa-2b treatment may have contributed to a reduced myelofibrosis transformation risk. Younger patients and those with low allele burden are reported to be more likely to have a molecular response to ropeginterferon alfa-2b [8]. Similarly, patients who achieved < 50% allele burden at week 52 had a lower baseline allele burden compared to those who did not (64.4% vs 91.5%) in this study. Additionally, the proportion of patients with CHR at week 52 was 100% (10/10 cases; one case could not be determined due to discontinuation) in patients who achieved < 50% allele burden at week 52. These results suggest that an allele reduction is associated with the CHR achievement. However, longer studies with larger populations are needed to confirm these results and to determine which patients are more likely to achieve a molecular response in Japanese patients with low-risk PV.

Although TEAEs related to ropeginterferon alfa-2b occurred in all patients, no treatment-related grade ≥ 3 TEAEs occurred. As in the original phase 2 study, the most common TEAE was alopecia [9]. One patient who discontinued treatment because of silent thyroiditis related to ropeginterferon alfa-2b had no history of thyroid dysfunction and was positive for anti-thyroid peroxidase antibodies and negative for anti-thyroglobulin antibodies at baseline [13]. Therefore, it is advisable to monitor thyroid function and thyroid antibodies both prior to and following ropeginterferon alfa-2b initiation.

Our results indicate that ropeginterferon alfa-2b can effectively reduce HCT, WBC counts, PLT counts, and *JAK2* V617F allele burden, reducing phlebotomy requirements, in Japanese patients with low-risk PV. Although other details (including clinical symptoms, duration, and phlebotomy frequency) were not recorded, and despite a relatively small sample size and short duration, our results support ropeginterferon alfa-2b as a treatment option for patients with low-risk PV who do not respond adequately to phlebotomy and low-dose aspirin.

### Supplementary Information

Below is the link to the electronic supplementary material.

Supplementary file1 (DOCX 60 KB)

## Data Availability

The data are available from PharmaEssentia upon reasonable request.
