# Supplementary material for: Efficacy and safety outcomes in Japanese patients with low-risk polycythemia vera treated with ropeginterferon alfa-2b
Source: Int J Hematol. 2024 Jul 1;120(2):151–6. doi: 10.1007/s12185-024-03804-1 (PMC11284189; doi:10.1007/s12185-024-03804-1)
Supplement: Supplementary file 1 — Supplementary file1 (DOCX 60 KB) [file 12185_2024_3804_MOESM1_ESM.docx]

**Electronic supplementary material**

**Supplemental Table 1.** Key inclusion criteria.

| **Key inclusion criteria** |
| --- |
| - Patients for whom the current standard of treatment is difficult to apply: - Younger patients for whom long-term treatment is anticipated. - Patients categorized as low risk, but for whom cytoreduction is recommended due to disease-related signs and symptoms (headache, dizziness, pruritus, night sweats, fatigue, erythromelalgia, vision disorders, scintillating scotoma, early satiety, and abdominal distension). - Patients with history of hydroxyurea treatment intolerance [modified European LeukemiaNet criteria]) [1]. - The following additional inclusion criteria were applied only to cytoreduction-naïve patients who were deemed to require cytoreductive treatment at baseline, defined as meeting one or more of the following criteria: - Patients with ≥1 previous well documented major cardiovascular polycythemia vera-related event. - Patients with poor tolerance of phlebotomy defined as a phlebotomy/procedure-related adverse event causing significant adverse impact on the patient and limiting ability to apply phlebotomy with the intention to keep hematocrit <45%. - Patients with frequent need of phlebotomy defined as ≥2 phlebotomies within the last month prior to participating the study. - Patients with platelet count >1,000 × 10^9^/L or WBC count > 10 × 10^9^/L at two measurements within the month prior to treatment start. - Patients with manifestation of disease-related signs and symptoms (e.g., headache, dizziness, pruritus, night sweats, fatigue, erythromelalgia, vision disorders, scintillating scotoma, early satiety, or abdominal distension). |

1. Barosi G, Birgegard G, Finazzi G, Griesshammer M, Harrison C, Hasselbalch H, et al. A unified definition of clinical resistance and intolerance to hydroxycarbamide in polycythaemia vera and primary myelofibrosis: Results of a European LeukemiaNet (ELN) consensus process. Br J Haematol. 2010;148:961–3.

**Supplemental Table 2.** Treatment-emergent adverse events related to ropeginterferon alfa-2b occurring in ≥2 patients with low-risk polycythemia vera.

|  | ***N* = 20** |
| --- | --- |
| Alopecia | 11 (55.0) |
| Influenza-like illness | 7 (35.0) |
| Alanine aminotransferase increased | 6 (30.0) |
| Fatigue | 6 (30.0) |
| Aspartate aminotransferase increased | 5 (25.0) |
| Diarrhea | 5 (25.0) |
| Beta-2 microglobulin urine increased | 4 (20.0) |
| Liver function test abnormal | 3 (15.0) |
| Myalgia | 3 (15.0) |
| Pyrexia | 3 (15.0) |
| Abdominal pain | 2 (10.0) |
| Anemia | 2 (10.0) |
| Anti-thyroid antibody-positive | 2 (10.0) |
| Arthralgia | 2 (10.0) |
| Hypothyroidism | 2 (10.0) |
| Injection site reaction | 2 (10.0) |
| Insomnia | 2 (10.0) |
| Malaise | 2 (10.0) |
| White blood cell count decreased | 2 (10.0) |

Data are *n* (%) and classified according to the Medical Dictionary for Regulatory Activities, v23.0.

**
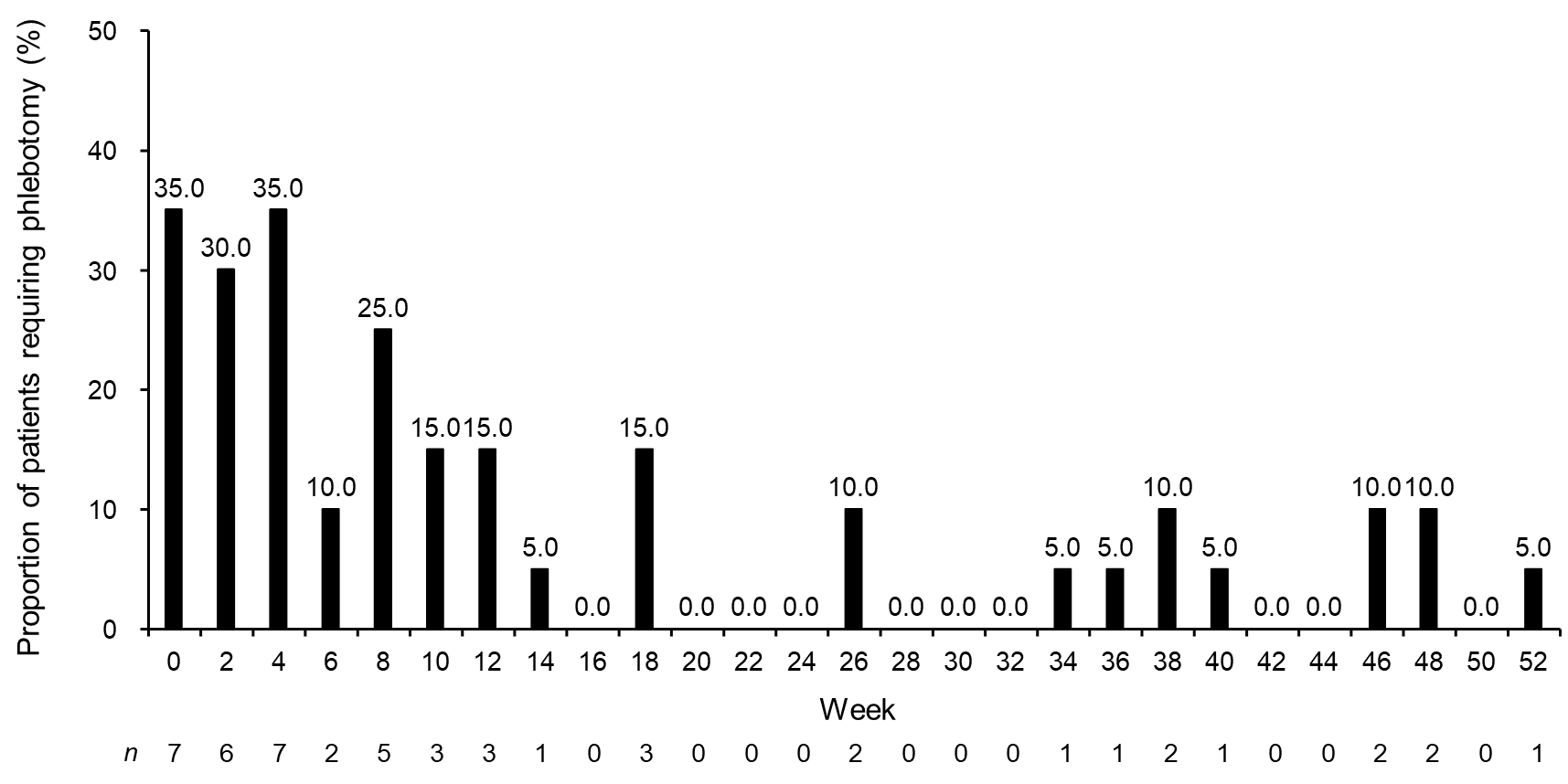
Supplemental Fig. 1.** Proportion of patients with low-risk polycythemia vera requiring phlebotomy over time.

*N* = 20
